# Expression changes of serum LINC00941 and LINC00514 in HBV infection‐related liver diseases and their potential application values

**DOI:** 10.1002/jcla.24143

**Published:** 2021-11-26

**Authors:** Juanjuan Chen, Dongling Tang, Huan Li, Pingan Zhang

**Affiliations:** ^1^ Laboratory Medicine Center Renmin Hospital of Wuhan University Wuhan China

**Keywords:** diagnosis, hepatitis B virus, LINC00514, LINC00941, liver diseases

## Abstract

**Background:**

Long non‐coding RNAs (LncRNAs) are considered as potential diagnostic markers for a variety of tumors. Here, we aimed to explore the changes of LINC00941 and LINC00514 expression in hepatitis B virus (HBV) infection‐related liver disease and evaluate their application value in disease diagnosis.

**Methods:**

Serum levels of LINC00941 and LINC00514 were detected by qRT‐PCR. Potential diagnostic values were evaluated by receiver operating characteristic curve (ROC) analysis.

**Results:**

Serum LINC00941 and LINC00514 levels were elevated in patients with chronic hepatitis B (CHB), liver cirrhosis (LC), and hepatocellular carcinoma (HCC) compared with controls. When distinguishing HCC from controls, serum LINC00941 and LINC00514 had diagnostic parameters of an AUC of 0.919 and 0.808, sensitivity of 85% and 90%, and specificity of 86.67% and 56.67%, which were higher than parameters for alpha fetal protein (AFP) (all *p *< 0.0001). When distinguishing HCC from LC, CHB, or LC from controls, the combined detection of LINC00941 or LINC00514 can significantly improve the accuracy of AFP test alone (all *p *< 0.05).

**Conclusions:**

LINC00941 and LINC00514 were increased in the serum of HBV infection‐associated liver diseases and might be independent markers for the detection of liver diseases.

## Graphical abstract

Long non‐coding RNA (lncRNA) is a promising serum biomarker for disease diagnosis. However, literature on the diagnostic value of the lncRNA for liver diseases, especially hepatocellular carcinoma (HCC), is scant. The present study is to detect the expression levels of LINC0094 and LINC00514 in hepatitis B virus (HBV) infection‐related liver diseases and evaluate their potential application in the diagnosis of liver diseases. Our results showed that serum LINC0094 and LINC00514 levels were significantly elevated in patients with chronic hepatitis B (CHB), liver cirrhosis (LC), and hepatocellular carcinoma (HCC) compared with healthy controls, and the combined detection with alpha fetal protein (AFP) can significantly improve the diagnostic efficiency of liver diseases.

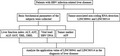

## 1 INTRODUCTION

Liver cancer is one of the most common human cancers worldwide, and also one of the most important causes of individual death caused by cancer, and its morbidity and mortality rank 6th and 2nd among all malignant tumors, respectively.^1^ According to the latest global cancer statistics in 2018, there are about 840,000 new cases of liver cancer each year, with an incidence rate of about 4.7%; and about 780,000 new liver cancer deaths occur every year, with a mortality rate of 8.2%.^2^ China is one of the countries with a high incidence of liver cancer. According to the pathologic characteristics of tissues, hepatocellular carcinoma (HCC) accounts for more than 90% of all liver cancers, and ranks fourth and third in incidence and mortality among all malignant tumors; in addition, compared with females, males have a higher incidence and poorer prognosis.^3^, ^4^ HCC is mainly caused by hepatitis virus infection, alcohol abuse, non‐alcoholic steatohepatitis, toxin exposure, and metabolic syndrome.^5^ Chronic infection of hepatitis B virus (HBV) and aflatoxin exposure are the main pathogenic factors of HCC in China, and HCC caused by chronic HBV infection accounts for more than 80% of all HCC.^6^ In addition to liver ultrasound, serum alpha fetal protein (AFP) detection is the main method for extensive screening of HCC in China. However, due to its low sensitivity and specificity, the detection rate of HCC is not high, so that many HCC patients miss the optimal surgical period. Therefore, it is very important to explore novel and effective serum markers to improve the detection rate and prognosis of HCC.

Non‐coding RNA is currently a research hotspot in the field of molecular biology. Although it does not have the function of encoding protein, it plays an important role in epigenetic, transcription, and post‐transcriptional levels. More and more lncRNAs have been found to be involved in the occurrence and development of a variety of tumors, and can be used for tumor diagnosis and prognosis monitoring. Studies have reported that the expression of LINC00941 is increased in pancreatic cancer, colorectal cancer, lung cancer, etc.^7^, ^8^, ^9^, ^10^ It can promote tumor progression through a variety of signaling pathways, such as proliferation, metastasis, invasion, and so on.^7^, ^8^, ^9^, ^11^ It can also be used as a diagnostic or prognostic marker for gastric cancer, lung cancer, head and neck squamous cell carcinoma, and other tumors^12^, ^13^, ^14^ and could predict the recurrence‐free survival and overall survival of HCC.^15^ On the other hand, it has been reported that LINC00514 is highly expressed in the tissues and cells of breast and pancreatic cancer and can promote tumor occurrence and development by regulating related microRNAs.^16^, ^17^ However, so far, no studies have reported their diagnostic value in HCC.

In this research, we detected the expression of LINC00941 and LINC00514 in healthy controls and patients with HBV infection‐related liver disease and assessed its correlation with basic characteristics of patients with HCC. The diagnostic values of LINC00941 and LINC00514 in liver diseases were also analyzed by receiver operating characteristic curve (ROC).

## 2 MATERIAL AND METHODS

### 2.1 Study population

All subjects were collected from inpatient department of Renmin Hospital of Wuhan University from November 2019 to January 2020. A total 147 subjects were divided into HBV‐associated HCC group (40 cases), HBV‐associated liver cirrhosis (LC) group (40 cases), chronic hepatitis B (CHB group) (37 cases). In addition, 30 normal males who received physical examination in our hospital during the same period were selected as the normal control group. The ages of all subjects ranged from 30 to 80 years old, and there was no significant difference in age among all groups. The diagnosis of HCC and LC patients was in accordance with the guidelines of liver Disease Society of Chinese Medical Association and Infectious Diseases Society of China, and they were confirmed by liver biopsy, X‐ray computed tomography or MAGNETIC resonance imaging. HCC and LC caused by other causes, as well as other infectious diseases, malignant tumors, and autoimmune diseases, were excluded. The diagnosis of CHB patients meets the Diagnostic Criteria for Chronic Hepatitis B (2015 edition).

This research was approved and reviewed by the Medical Ethics Review Committee of Renmin Hospital of Wuhan University. All participants agreed and signed the written informed consent in accordance with policies of the hospital Ethics Committee.

### 2.2 Sample collection

All vacuum blood collection tubes were purchased from BD Company. The yellow head tube to promote blood coagulation and the pearl‐white head tube to promote blood coagulation of all subjects were collected on an empty stomach for more than 8 h in the morning. The yellow head tube blood was used for routine biochemical indexes, AFP, LINC00941, and LINC00514 detection, and the pearl‐white head procoagulant blood was used for HBV DNA detection. After collection, the blood was placed at room temperature for 15min. When the blood was completely coagulated, the serum was separated after centrifugated at 3500 r/min for 5 min for use.

### 2.3 Laboratory analysis

Automatic biochemical analyzer (Siemens, Germany) and supporting analysis reagents were used to detect serum alanine aminotransferase (ALT, normal reference range: 9–50 U/L), aspartate aminotransferase (AST, normal reference range: 15–40 U/L), alkaline phosphatase (ALP, normal reference range: 45–125 U/L), gamma‐glutamyl transferase (GGT, normal reference range: 10–60 U/L), albumin (ALB, normal reference range: 40–55 g/L), total bilirubin (TBIL, normal reference range: 0–23 μmol/L), and direct bilirubin (DBIL, normal reference range: 0–8 μmol/L). The real‐time fluorescent quantitative PCR instrument (ABI ViiA7, USA) was used to detect the HBV DNA load level, and the kit was produced by Shanghai Fosun Long March Medical Science Co., Ltd. Automatic immunoluminescence analyzer (Siemens, Germany) and supporting reagents were used to detect serum AFP.

### 2.4 Serum LINC00941 and LINC00514 extraction and qRT‐PCR analysis

The serum LINC00941 and LINC00514 were extracted with Beijing Biotech (blood RNA extraction and adsorption column type) kit. The above two non‐coding RNAs were quantified by fluorescence quantitative PCR using the reverse transcription and quantitative PCR kits (RR036A and RR091A) of Takara, Japan, and GAPDH as the housekeeping gene. Reverse transcription was performed at 37℃ for 15 min and 85℃ for 5S; The RT‐PCR procedure was 95℃ for 30 s and the number of cycles was 1; 95℃ for 5 s, 64℃ for 30 s, and the number of cycles is 40. Primer sequences are as follows: LINC00941: forward primer CAAGCAACCGTCCAACTACCAGACA, reverse primer AAATCAAGAGCCCAAACATTGTGAA; LINC00514: forward primer CAACCAGGTGCTGGGGACAG, reverse primer GACCTCAAGTGATCCGCCCG; GAPDH: forward primer GGAGCGAGATCCCTCCAAAAT, reverse primer GGCTGTTGTCATACTTCTCATGG; and primer concentration was 10 μmol/L. The relative quantitative results were expressed by 2^−△CT^ method.

### 2.5 Statistical analysis

SPSS 20.0 and MedCalc 15.2.2 software were used for statistical analysis of the data, and GraphPad Prism 6 software was used for drawing. Kolmogorov‐Smirnov (K‐S) test was used to evaluate the normality of each group of data. The quantitative data complying with normal distribution were represented by mean ± SEM, and one‐way analysis of variance (ANOVA) was used for comparison among multiple groups. LSD test was used for homogeneity and Tamhane'ST2 test was used for non‐homogeneity. The quantitative data that did not obey the normal distribution was represented by the median and interquartile ranges, and the Mann‐Whitney test was used to analyze the corresponding data. Pearson's correlation was used to evaluate the correlation among all indicators. ROC curve was used to analyze the diagnostic value of LINC00941, LINC00514, and AFP for HCC. All data were analyzed by two‐sided test, and *p *< 0.05 was considered as statistically significant.

## 3 RESULTS

### 3.1 Characteristics of healthy controls and patients with HBV infection‐related liver disease

The main characteristics of all the study population are summarized in Table 1. First, there was no statistically significant difference in age between the groups. Regarding the liver biochemical indicators ALT, AST, ALP, GGT, ALB, TBIL, DBIL, the differences between the four groups were statistically significant (all *p *< 0.0001). For the level of HBV DNA, the CHB group was significantly higher than the LC group and the HCC group (all *p *< 0.0001). In the analysis of serum tumor markers, result showed that the AFP level of HCC patients was obviously increased than that of the control group, CHB and LC groups, and the difference was statistically significant.

**TABLE 1:** Basic biochemical data characteristics of controls and patients with HBV infection‐related liver disease

Variables	Controls (n = 30)	CHB (n = 37)	LC (n = 40)	HCC (n = 40)	*p* value
Age (years)	45.67 ± 15.02	41.86 ± 13.11	52.05 ± 11.75	54.77 ± 11.25	0.200
ALT (U/L)	19.5 (15–27)	102 (45.5–308.5)	44 (26.5–88)	35 (25–62)	＜0.001
AST (U/L)	20.5 (18–24)	60 (36–133)	48 (34.3–106.5)	38 (26–110)	＜0.001
ALP (U/L)	70.6 (63.5–80.6)	94.2 (74.9–117.2)	100.9 (73.5–145.8)	123.4 (84.1–197.2)	＜0.001
GGT (U/L)	18 (14.8–28)	51 (24.5–141.5)	70.5 (36.8–104.8)	114 (56–230)	＜0.001
ALB (g/L)	43.75 (42.98–45.03)	42.80 (38.00–46.05)	32.75 (28.48–35.88)	39.10 (34.70–41.40)	＜0.001
TBIL (μmol/L)	12.10 (9.20–14.65)	21.10 (14.95–40.05)	26.6 (14.70–51.08)	16.60 (11.80–31.40)	＜0.001
DBIL (μmol/L)	3.75 (3.20–4.42)	7.10 (5.30–22.30)	11.55 (5.93–33.02)	5.80 (4.10–34.72)	＜0.001
HBV DNA (log_10 IU_/ml)	/	4.33 (2.80–6.60)	3.58 (1.78–5.01)	1.63 (2.93–4.42)	＜0.001
AFP(ng/ml)	3.15 (2.20–4.83)	7.90 (2.50–25.60)	11.65 (3.00–77.30)	30.10 (6.10–1186.00)	＜0.001

Note

Data are presented as means (SD) or median (interquartile range) or percentage.

Abbreviations: AFP, alpha fetoprotein; ALB, albumin; ALP, alkaline phosphatase; ALT, alanine aminotransferase; AST, aspartate aminotransferase; CHB, Chronic hepatitis b; DBIL, direct bilirubin; GGT, gamma‐glutamyl transpeptidase; HBVDNA, hepatitis b virus deoxyribonucleic acid; HCC, hepatocellular carcinoma; LC, liver cirrhosis; TBIL, total bilirubin.

### 3.2 Comparison of serum LINC00941 and LINC00514 levels in controls, CHB, LC and HCC

**FIGURE 1:**
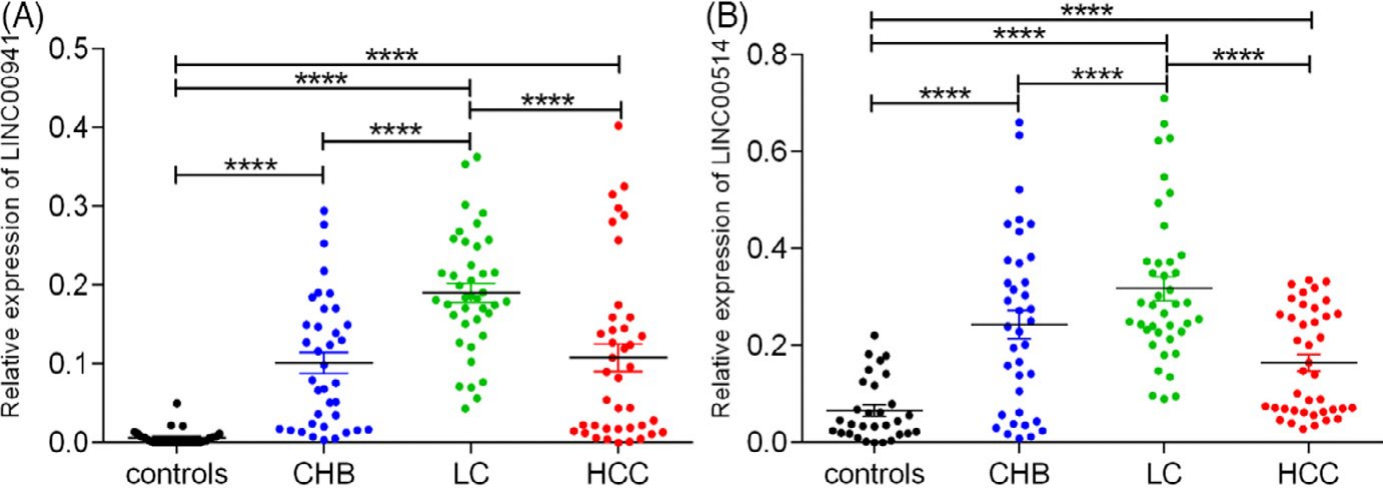
Serum LINC00941 and LINC00514 expression in patients with hepatocellular carcinoma (HCC), patients with liver cirrhosis, patients with HCC and healthy controls. ^****^ *p *< 0.0001 represents significant difference between two groups

Furthermore, we used the LSD test to evaluate the serum levels of LINC00941 and LINC00514 in the controls, CHB, LC and HCC groups. As shown in Figure 1A, serum LINC00941 levels in the CHB, LC and HCC groups were significantly higher than those in the control group (all *p *< 0.0001), and compared with the patients with LC, the level of serum LINC00941 in CHB and HCC groups were obviously decreased (all *p *< 0.0001). Compared to the controls, the serum LINC00514 level was significantly increased in patients with CHB, LC and HCC (all *p *< 0.0001); and the serum LINC00514 level was significantly lower in the CHB group and the HCC group than that of LC group (all *p *< 0.0001, Figure 1B).

### 3.3 Relationship between serum LINC00941 and LINC00514 expression and basic biochemical indexes in patients with HCC

To further evaluate whether serum LINC00941 and LINC00514 expression are related to the liver function, HBV viral load and AFP level of HCC patients, We analyzed the correlation between the high and low expression of two lncRNAs and the above indexes. The results showed that serum LINC00941 and LINC00514 had no correlation with liver function index, HBV viral load and AFP (Table 2).

**TABLE 2:** The association between the relative expression of LINC00941 and LINC00514 in serum of HCC patients and basic clinical data

Variables	Patients (n = 40)	LINC00941 expression levels		*p* value	Patients (n = 40)	LINC00514 expression levels		*p* value
		Low expression	High expression			Low expression	High expression	
<54	16	7	9		16	9	7	
≥54	24	15	9		24	12	12	
ALT								0.412
<51	28	13	15	0.496	33	16	17	
≥51	12	9	3		7	5	2	
AST								0.273
<76	26	13	13	0.386	26	12	14	
≥76	14	9	5		14	9	5	
ALP				0.919				0.427
<160	27	15	12		27	13	14	
≥160	13	7	6		13	8	5	
GGT				0.919				0.906
<167	27	15	12		27	14	13	
≥167	13	7	6		13	7	6	
ALB				0.131				0.218
<38.2	17	7	10		17	7	10	
≥38.2	23	15	8		23	14	9	
TBIL				1.000				0.421
<38.6	33	18	15		33	16	17	
≥38.6	7	4	3		7	5	2	
DBIL				0.673				0.186
<20.5	34	18	16		34	16	18	
≥20.5	6	4	2		6	5	1	
HBV DNA				0.564				0.141
<500	27	14	13		27	12	15	
≥500	13	8	5		13	9	4	
AFP				0.151				0.301
<200	14	12	2		24	11	13	
≥200	16	10	6		16	10	6	

Note

The data are divided on average.

Abbreviations: AFP, alpha fetoprotein; ALB, albumin; ALP, alkaline phosphatase; ALT, alanine aminotransferase; AST, aspartate aminotransferase; DBIL, direct bilirubin; GGT, gamma‐glutamyl transpeptidase; HBVDNA, hepatitis b virus deoxyribonucleic acid; HCC, hepatocellular carcinoma; TBIL, total bilirubin.

### 3.4 Serum levels of LINC00941, LINC00514 and AFP at different HCC stages and liver function grades

**FIGURE 2:**
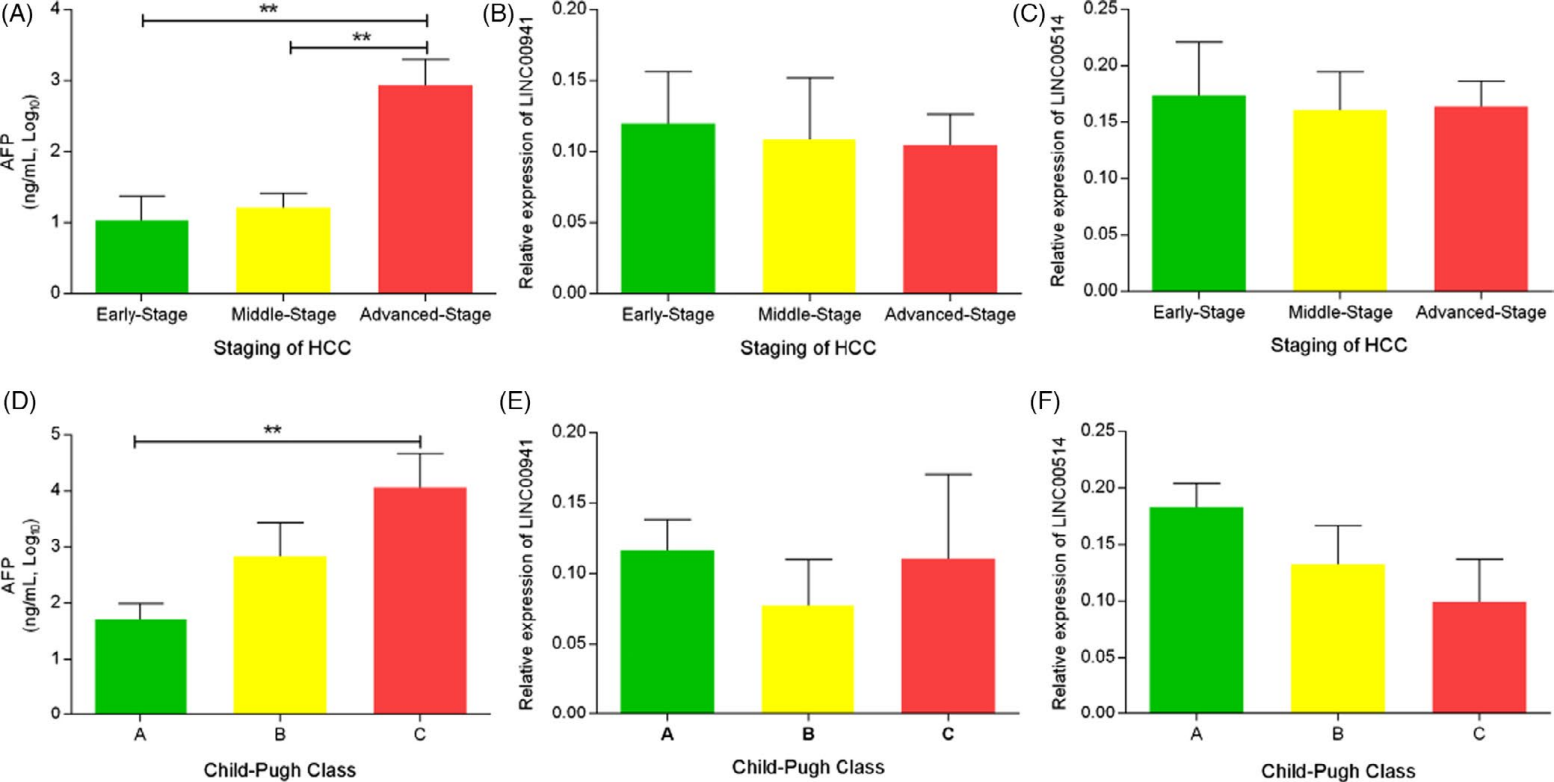
Serum concentration of LINC00941, LINC00514 and alpha fetoprotein according to Barcelona Clinic Liver Cancer and Child‐Pugh class in patients with hepatocellular carcinoma. ^**^ *p *< 0.01 represents significant difference between two groups

To assess the changes of LINC00941, LINC00514, and AFP levels in the progression of HCC, patients with HCC were divided into early, middle and advanced stages, according to the Barcelona Clinic Liver Cancer (BCLC). We found that serum level of AFP in advanced‐stage HCC was significantly higher than early stage and middle stage (all *p *< 0.01, Figure 2A). However, the levels of LINC00941 and LINC00514 showed no difference in different stages of HCC but showed a gradual downward trend (Figure 2B,C). According to Child‐Pugh class, patients with HCC were divided into class A, B, and C. The results indicated that the AFP level of class C patients was obviously higher than that of class A patients, while the LINC00941 and LINC00514 levels showed no difference in different liver function grades, and LINC00514 showed a trend of progressive decrease in different grades (Figure 2D–F).

### 3.5 Diagnostic value of serum LINC00941, LINC00514 and AFP

**FIGURE 3:**
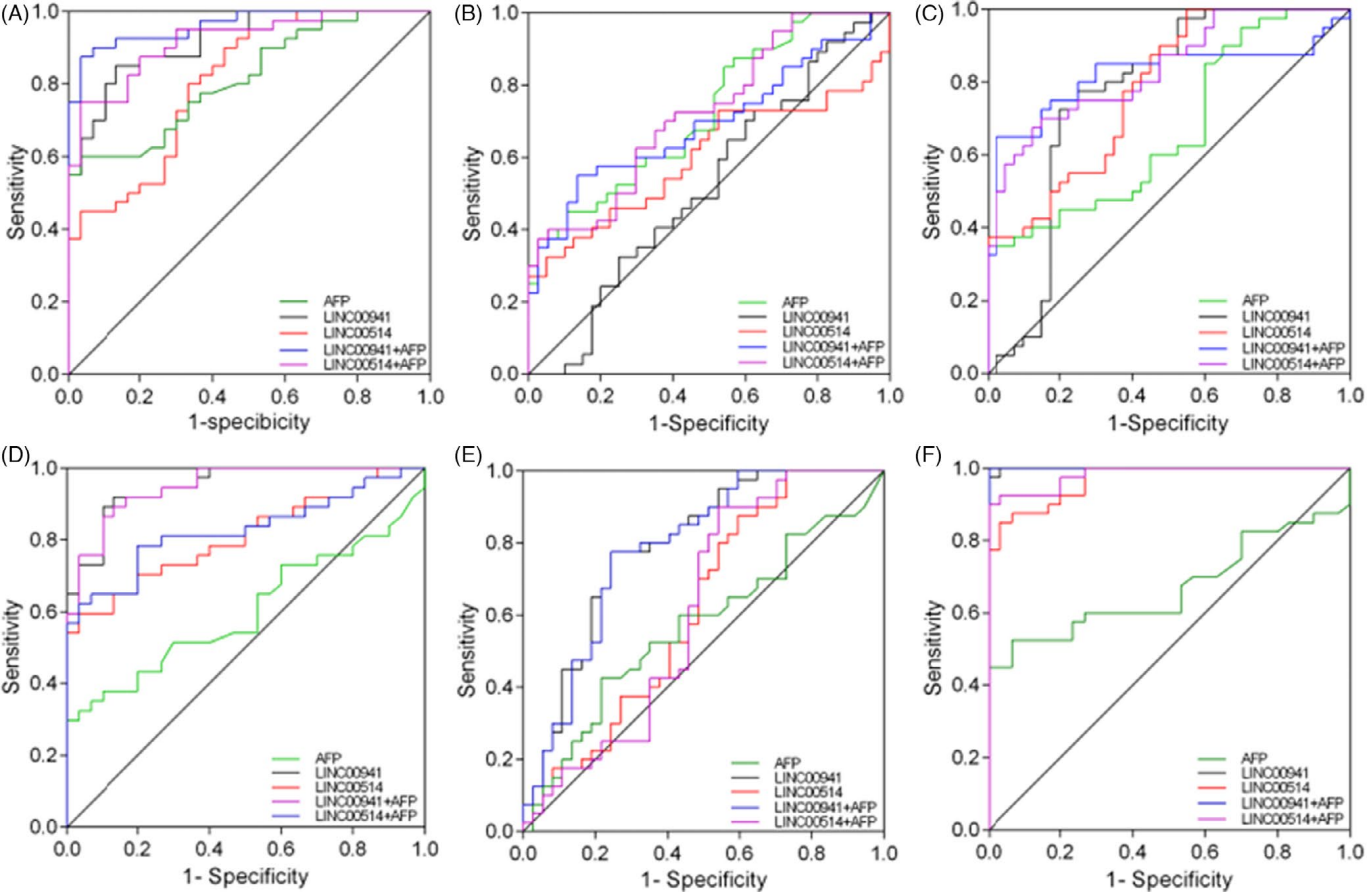
ROC curves of serum LINC00941, LINC00514 and alpha fetoprotein (AFP) in the differential diagnosis of hepatocellular carcinoma (HCC). (A) HCC versus controls. (B) HCC versus chronic hepatitis B (CHB). (C) HCC versus liver cirrhosis (LC). (D) CHB versus controls. (E) LC versus CHB. (F) LC versus controls

To explore whether serum LINC00941 and LINC005141 can be used as novel biomarkers for HCC, we evaluated the diagnostic value of LINC00941 and LINC00514 using the ROC curve model, and used AFP as a reference. The results showed that the sensitivity and specificity of LINC00941 and LINC00514 in distinguishing HCC from healthy controls were 85.00%, 90% and 86.67%, 56.67%, respectively (Figure 3A). In addition when combined with AFP, both LINC00941 and LINC00514 could improve the accuracy of HCC diagnosis (0.962 vs. 0.815 and 0.918 vs. 0.815, *p *< 0.001 and *p *< 0.01). LINC00941 and LINC00514 showed no significant advantage in distinguishing HCC from CHB compared to AFP (Figure 3B). When distinguishing HCC from LC, LINC00941 and LINC00514 combined with AFP significantly improved the accuracy of HCC diagnosis (0.820 vs. 0.668 and 0.835 vs. 0.668, all *p *< 0.01, Figure 3C). Compared with AFP alone, LINC00941 and LINC00514 alone or in combination with AFP improved sensitivity and accuracy in the diagnosis of CHB and LC (used healthy controls as control group, Figure 3D,F). When differentiating LC and CHB, LINC00941 alone or in combination with AFP significantly improved the sensitivity and accuracy of LC diagnosis compared with AFP alone (all *p *< 0.01, Figure 3E) (Table 3).

**TABLE 3:** Differential diagnostic efficacy of serum LINC00941, LINC00514, AFP and the combination

Group	Marker	AUC	*p* value	95% CI	SEN (%)	SPE (%)
HCC vs controls	LINC00941	0.919	<0.0001	0.859–0.980	85	86.67
	LINC00514	0.808	<0.0001	0.707–0.908	90	56.67
	AFP	0.815	<0.0001	0.718–0.911	60	96.67
	LINC00941+AFP	0.962^###^	<0.0001	0.923–1.000	87.5	96.67
	LINC00514+AFP	0.918^#**^	<0.0001	0.856–0.980	75	96.67
HCC vs CHB	LINC00941	0.521	0.752	0.404–0.636	85	2.7
	LINC00514	0.599	0.134	0.481–0.709	95	32.43
	AFP	0722^#^	0.001	0.608–0.818	45	89.19
	LINC00941+AFP	0.692	0.004	0.576–0.792	55	86.49
	LINC00514+AFP	0.724^*^	0.001	0.611–0.820	37.5	97.3
HCC vs LC	LINC00941	0.766	<0.0001	0.685–0.854	75	75.5
	LINC00514	0.781	<0.0001	0.674–0.865	45	100
	AFP	0.668	0.0057	0.554–0.770	35	100
	LINC00941+AFP	0.820^#^	<0.0001	0.718–0.897	65	97.5
	LINC00514+AFP	0.835^#^	<0.0001	0.735–0.909	67.5	87.5
CHB vs controls	LINC00941	0.950^####^	<0.0001	0.867–0.988	89.19	90
	LINC00514	0.808^##^	<0.0001	0.694–0.894	59.46	96.67
	AFP	0.600	0.1538	0.473–0.718	29.73	100
	LINC00941+AFP	0.949^####^	<0.0001	0.866–0.988	86.49	90
	LINC00514+AFP	0.826^##^	<0.0001	0.714–0.908	62.16	96.67
LC vs CHB	LINC00941	0.791^##^	<0.0001	0.684–0.876	77.5	75.68
	LINC00514	0.616	0.0807	0.498–0.724	87.5	40.54
	AFP	0.568	0.3058	0.450–0.680	42.5	78.38
	LINC00941+AFP	0.785^##^	<0.0001	0.677–0.871	77.5	75.68
	LINC00514+AFP	0.612	0.0990	0.494–0.721	90	45.95
LC vs controls	LINC00941	0.999^####^	<0.0001	0.947–1.000	97.5	100
	LINC00514	0.967^####^	<0.0001	0.894–0.995	85	96.67
	AFP	0.670	0.0101	0.547–0.777	52.5	93.33
	LINC00941+AFP	1.000^####^	<0.0001	0.949–1.000	100	100
	LINC00514+AFP	0.983^####^	<0.0001	0.918–0.999	90	100

Note

Compared with LINC00514, ^*^ *P* < 0.01, ^**^ *P* < 0.01; compared with AFP, ^#^ *P* < 0.05, ^##^ *P* < 0.01, ^###^ *P* < 0.001, ^####^ *P* < 0.0001.

Abbreviations: AFP, alpha fetoprotein; CHB, Chronic hepatitis B; HCC, hepatocellular carcinoma; LC, liver cirrhosis; SEN, sensitivity; SPE, specificity.

## 4 DISCUSSION

The prevalence of liver cancer is related to its many pathogenic factors and complex pathogenesis, which also means that it is difficult to diagnose with a single biomarker. Therefore, the combined detection of multiple biomarkers is of great significance to improve the detection rate of liver cancer. At present, a variety of biomarkers have been applied to the diagnosis of HCC, including AFP, miRNA, specially expressed genes, lncRNA, etc. Among them, lncRNA is a new research direction and hotspot in recent years.

In previous studies, various lncRNAs have been studied in liver cancer. For example, H19 is the first non‐coding RNA reported to be abnormally expressed in HCC.^18^, ^19^ Unfried et al. reported that the expression of NIHCOLE was related to the poor prognosis and survival of patients with HCC, and the inhibition of NIHCOLE expression in HCC cells may lead to limited cell proliferation and increased apoptosis rate through the accumulation of DNA damage.^20^ Research by Peng et al. showed that the expression of LINC00511 was higher in HCC tissues, and the mechanism study revealed that the invasion pseudopodia and exosomal secretion induced by LINC00511 were involved in tumor progression.^21^ Yin et al. found that LINC01133 expression in HCC tissues was elevated, and can predict the poor prognosis of HCC patients; in‐vitro studies showed that overexpression of LINC01133 can promote the proliferation and aggressive phenotype of HCC cells, and promote tumor growth and lung metastasis in vivo, while knockdown LINC01133 had the opposite effect.^22^ In addition, studies have also reported that lncRNA plays an important role in the chemotherapy resistance of HCC. The study of Ma et al. found that the expression of LINC01134 was upregulated after treatment with oxaliplatin (OXA), and the higher expression of LINC01134 was related to the poor treatment effect of OXA; mechanistic studies have shown that the LINC01134/SP1/p62 axis modulated OXA resistance by changing cell viability, apoptosis and mitochondrial homeostasis in vitro and in vivo, suggesting that the LINC01134/SP1/p62 axis may be a promising strategy to overcome OXA chemotherapy resistance.^23^ These findings suggest that lncRNAs play an important role in the occurrence and development, treatment, and prognosis monitoring of HCC.

In our study, we detected the expression levels of LINC00941 and LINC00514 in the serum of controls, CHB, LC, and HCC patients, and found that compared with the healthy control group, the serum levels of LINC00941 and LINC00514 in the CHB, LC and HCC groups were significantly increased. The levels of LINC00941 and LINC00514 in the LC group were significantly higher than those in the CHB and HCC groups.

In order to investigate whether the expression levels of LINC00941 and LINC00514 are related to the basic biochemical parameters of HCC patients, we divided the expression levels of LINC00941 and LINC00514 into high and low levels, and the results showed that the expression levels of LINC00941 and LINC00514 were not related to the basic biochemical parameters of HCC patients. Further, we performed tumor staging and liver function grading for HCC patients, and compared the level of AFP, LINC00941, and LINC00514 in different stages and grades, and found that AFP levels in advanced HCC patients were significantly higher than those in early and middle‐stage HCC patients, LINC00941 and LINC00514 showed no significant difference in different HCC stages, but showed a trend of gradual decrease with the development of HCC. The level of AFP in class C HCC patients was significantly higher than that in class A HCC patients, and the level of LINC00514 gradually decreased with the deterioration of liver function in HCC patients.

In order to evaluate the value of LINC00941 and LINC00514 as diagnostic markers for HCC, we detected the serum levels of LINC00941, LINC00514, and AFP in controls, CHB, LC and HCC populations, respectively, and evaluated the diagnostic value of LINC00941 and LINC00514 for HCC, LC, or CHB. In the current small sample study, the sensitivity of serum LINC00941 and LINC00514 in differential diagnosis of normal control and HCC patients were 85% and 90%, specificity was 86.67% and 56.67%, and the AUC reached 0.919 and 0.808, indicating that serum LINC00941 and LINC00514 had good diagnostic efficacy in the diagnosis of HCC; and when combined with AFP, they can significantly improve the sensitivity and accuracy of AFP diagnosis (87.5% vs. 60%, 75% vs. 60% and 0.962 vs. 0.815, 0.918 vs. 0.815). LINC00941 and LINC00514 did not show obvious advantages in the differential diagnosis of HCC and CHB. When used to diagnose HCC and LC, LINC00941 and LINC00514 both show good specificity and accuracy (75.5%, 100% and 0.766, 0.781), LINC00941 had a sensitivity of 75%, while LINC00514 had a poor sensitivity of only 45%; when combined with AFP, LINC00941 and LINC00514 can significantly improve the sensitivity and accuracy of AFP detection (65% vs. 35%, 67.5% vs. 35% and 0.820 vs. 0.668, 0.835 vs. 0.668). When used to distinguish between CHB and controls or LC and controls, LINC00941 and LINC00514 alone or in combination with AFP increased sensitivity and accuracy in the diagnosis of CHB and LC compared with AFP alone. When distinguishing between LC and CHB, compared with AFP alone, LINC00941 alone or combined with AFP can significantly improve the sensitivity and accuracy of LC diagnosis.

## 5 CONCLUSIONS

LINC00941 and LINC00514 were upregulated in HBV infection‐associated liver disease. Their abnormal expression can be used as an independent marker for the diagnosis of liver diseases. However, the relevant mechanisms and effects are still unclear. Moreover, this research is limited by sample size. Hence, further studies are needed.

## CONFLICT OF INTEREST

The authors have no conflicts of interest.

## AUTHOR CONTRIBUTION

PZ, JC, and DT proposed the concept of the work. JC carried out most of the experimental work and wrote the paper. HL provided critical reviews in order to promote the manuscript. All authors read and approved the final manuscript.

## Data Availability

The data that support the findings of this study are available from the corresponding author upon reasonable request.
